# Tidal tail identification and detailed analysis of the open star cluster King 13 using Gaia DR3 and 2MASS

**DOI:** 10.1038/s41598-025-96923-6

**Published:** 2025-05-23

**Authors:** Nasser M. Ahmed, Mohamed S. Darwish

**Affiliations:** National Research Institute of Astronomy and Geophysics (NRIAG), 11421 Helwan, Cairo Egypt

**Keywords:** Star cluster, Gaia DR3, 2Mass, CMD, Parallax, Proper motion, Distance, Membership, Chemistry, Astronomy and planetary science, Astronomy and astrophysics, Planetary science, Space physics

## Abstract

We present a comprehensive study of the young open cluster King 13 using photometric and astrometric data from Gaia DR3 and 2MASS. Our analysis refines the cluster’s fundamental parameters, including its structure, kinematics, and evolutionary status. *To assess membership, we employed the* pyUPMASK *Python package with the HDBSCAN algorithm. The primary emphasis of this study is our new approach to assign a membership probability at each radius, rather than applying a single value to the entire cluster. These probabilities are calculated based on the number of stars deduced from the King model*. This revealed a dense core with an elongated halo aligned with the cluster’s tangent velocity. Cluster orbital analysis suggests the cluster moves in the Galactic plane toward the Galactic center, with its tidal tail aligned with orbital motion-likely due to Galactic tidal effects. We identified 1571 $\pm$ 41 member stars with a total mass of 2658.4$\pm$ 61.5 M$\odot$. The mass function (MF) for the cluster has been constructed using a step function with two power lows, $\alpha _1$ and $\alpha _2$, rather than the single power low suggested by Salpeter. In this cluster, the $\alpha _1$ and $\alpha _2$ are found to be -3.7$\pm$0.4 and 2.3 $\pm$0.15 , respectively. . The cluster’s physical parameters were derived using PARSEC stellar isochrones, estimating an age of 310$\pm$ 28 Myr and a relaxation time of 134 $\pm$ 13 Myr, indicating dynamical stability. The proper motions ($\mu {\alpha } \cos \delta$, $\mu _{\delta }$) and parallax ($\varpi$) were measured as -2.64 $\pm$ 0.36 mas yr$^{-1}$, -0.89 $\pm$ 0.25 mas yr$^{-1}$, and 0.245 $\pm$ 0.05 mas, respectively. The corresponding distance of the cluster, derived from the parallax, is 4082 $\pm$ 231 pc. The derived distance modulus is 13.11$\pm$ 1.03 mag ( 4187$\pm$ 262 pc), with color excess values of 1.17 $\pm$ 0.07 mag (Gaia) and 0.44 $\pm$ 0.03 mag (2MASS), further validating our results. Additionally, 46 member stars with radial velocity data allowed us to compute the cluster’s orbit using the galpy package. Our findings highlight the presence of a tidal tail directed toward the center of the Galaxy and underscore the role of Galactic tidal forces in shaping King 13’s morphology, reinforcing its importance in the evolution of open clusters.

## Introduction

Open star clusters (OCs) are crucial for advancing our understanding of stellar evolution and the structure of galaxies. OCs, which are gravitationally bound groups of stars with similar ages and chemical abundances, are essential for deciphering the formation history of the Milky Way’s disc. They consist of stars that originated under nearly identical physical conditions and within a short time frame, making them excellent tracers of the evolving conditions of the interstellar medium. They typically contain a few dozen to several thousand stars at comparable distances. OCs are particularly useful for studying the structure, kinematics, and various properties of the Milky Way (e.g.^1–4^). In recent years, OCs have been the subject of numerous studies. They are frequently employed to investigate various properties of the Galactic disk, including the spiral arms of the Milky Way^5–7^, stellar structure, and the process of star formation (e.g.^8–10^). Additionally, OCs are pivotal in examining chemical homogeneity and the age-metallicity relation^11–17^. They are distributed throughout the Milky Way disk and exhibit a wide range of ages, from less than 100 Myr to approximately 8 Gyr (e.g.^3,17–19^). The distribution of key physical parameters such as mass, age, and size plays a significant role in their formation and evolution (for a recent review, see^10^). Essential astrophysical parameters of OCs, including metallicity, color excess, age, extinction, and distance, can be determined from color-magnitude diagrams (CMDs) by comparing observed data with stellar models, such as isochrones.

A notable characteristic of OCs is the presence of tidal tails. These tidal tails in OCs are stellar structures that extend outward from the main body of the cluster, formed as a result of the gravitational interactions between the cluster and the Galactic potential or giant molecular cloud^20^. In young OCs (with ages $\lesssim$ 100 Myr), these structures may result from the remnants of the giant molecular clouds (GMCs) from which the clusters formed or from rapid gas expulsion (e.g.,^21,22^). In older clusters (ages $\gtrsim$ 100 Myr), mechanisms such as two-body relaxation or external forces, such as disk shocks, are likely responsible for the stripped stars found in tidal tails^23^. Several young OCs have been observed with tail-like structures, such as, IC 2391, IC 2602, NGC 2451A, NGC 2547^21^. Additionally, an increasing number of older OCs have been found with tidal tails, such as Hyades^24–26^ and NGC 6774^22^. Recently,^27^ researched for tidal tail in Gaia EDR3 data and they have found 72 open clusters having tidal tail. Simulations^20,26,28^ have improved our understanding of tidal tail properties. However, due to the lower star density compared to globular clusters and the varying velocity components between the cluster core and the tails^26^, identifying the stripped tail members in OCs remains challenging, particularly when hindered by parallax errors and the absence of radial velocity data.

*King*13 is an open star cluster located in the direction of Perseus spiral arm at $\alpha$ = 00$^h$ 10$^m$ 12.7$^s$ and $\delta$ = +61$^\circ$ 10’ 30” (J2000.0), which corresponds to Galactic coordinates of $l = 117.9821^\circ$ and $b = -01.2999^\circ$. Marx and Lehmann^29^ conducted a study of the cluster and derived a photometric UBV magnitudes for 80 stars, estimating the cluster’s distance to be $r = 1730 \pm 200$ pc. Using CCD UBV photometry for 1590 stars,^30^ determined that the cluster’s age was 300 Myr, with a distance of $3100 \pm 330$ pc and a color excess of $E(B-V) = 0.82 \pm 0.02$. Similarly,^31^ utilized CCD BV photometry for 2204 stars, finding the cluster’s age to be approximately 300 Myr, the distance to be $3670^{+137}_{-130}$ pc, and the color excess to be $E(B-V) = 0.86$ ± 0.13. Using the 2MASS database,^32^ estimated the distance to the cluster to be 2.11 ± 0.25 Kpc, while the age is found to be 1.00 ± 0.12 Gyr. Moreover,^33^ used Gaia DR2 and 2MASS data to estimate the cluster’s age and distance as 510 ± 60 Myr and 3.84 ± 0.15 kpc, respectively, while the number of cluster members was found to be 172. A recent study by^34^ reported that the cluster’s age is around 390 (8.595 ± 0.173) Myr and a distance of 3.1 ($\pm 0.062$) kpc. The tidal radius for the cluster is estimated to be 10.59 pc.

Despite these efforts, none of the previous studies have commented on cluster morphology phenomena, such as tidal tails. In addition, these studies lack information about the cluster’s orbit and detailed kinematics. The aim of this study is to provide a comprehensive photometric and astrometric analysis of King 13 using data from the Gaia DR3 and 2MASS database. This analysis enables us to derive key astrophysical parameters, including mass, age, metallicity, and radius, as well as astrometric parameters such as parallax and proper motion. Additionally, we explore the cluster’s dynamical evolution. Furthermore, the cluster’s morphology is examined, with a focus on identifying any features related to tidal tails.

The paper is organized as follows: Section Data outlines the criteria used to extract the initial data sample from Gaia DR3. The cluster’s structure, along with its radial density profile, is described in Section Cluster Structure and Radial Density Profile. In Section Proper Motion, Membership Determination, and Cluster Center, we present the astrometric analysis, cluster membership determination, and the identification of the cluster’s center. The photometric properties of the cluster members, as well as the detection of the tidal tail, are discussed in Sections The Color Magnitude Diagrams and Cluster Age. The cluster kinematics and dynamics analysis will be in section Cluster Kinematics and Dynamics. Finally, the main conclusions are summarized in Section Summary and Conclusions.

## **Data**

In this work, we employ two extensive and complementary datasets, Gaia DR3 and 2MASS, to analyze the open cluster King 13. These datasets provide a robust foundation for identifying cluster members, deriving astrophysical parameters, and studying the cluster’s structure and kinematics. Below, we provide an overview of the key features and relevance of these datasets to the present study:

### **Gaia DR3 data**

We retrieved data for King 13 from the Gaia DR3 catalog^35^. The dataset includes sky positions ($\alpha$, $\delta$), proper motions ($\mu _{\alpha }\cos \delta$, $\mu _{\delta }$), and parallaxes, with a limiting magnitude of $G = 21$ mag. Gaia DR3 provides astrophysical parameters for numerous celestial objects, derived from parallaxes, broad-band photometry, and mean radial velocity spectra. The parallax errors in Gaia DR3 range from 0.02 to 0.07 milliarcseconds (mas) for sources with $G \le 17$ mag, increasing to 0.5 mas at $G = 20$ mag and up to 1.3 mas at $G = 21$ mag. Similarly, proper motion errors range from 0.02 to 0.07 mas yr$^{-1}$ for $G \le 17$ mag, reaching 0.5 mas yr$^{-1}$ at $G = 20$ mag, and up to 1.4 mas yr$^{-1}$ at $G = 21$ mag. The catalog contains *G* magnitudes for approximately 1.806 billion sources, $G_{BP}$ magnitudes for around 1.542 billion sources, and $G_{RP}$ magnitudes for approximately 1.555 billion sources.

**Fig. 1:**
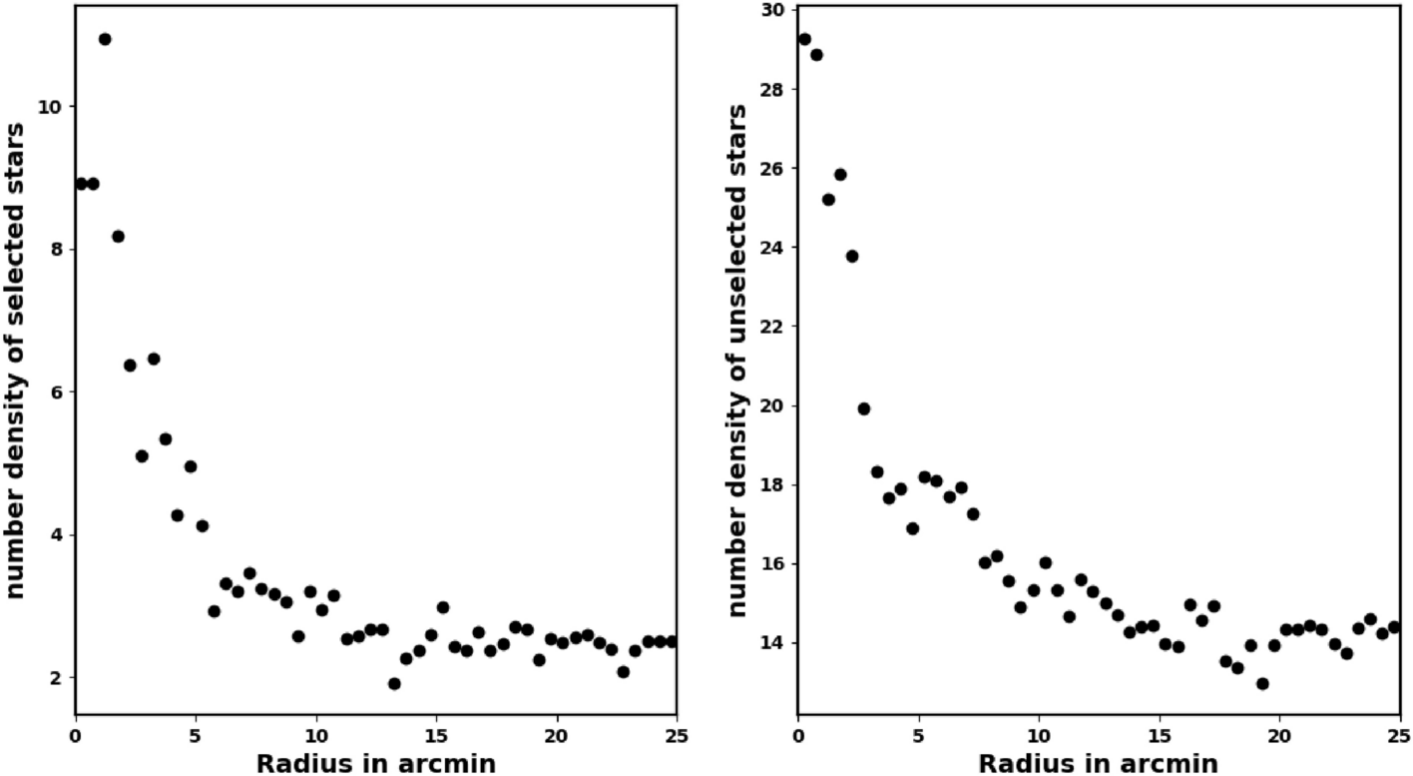
The left panel: the number stars density of selected stars, in case of the parallax condition $0.2 \le \varpi \ge 0.4$, while the right panel is RDP of unselected stars.

While processing Gaia data it is important to note that inaccurate data clipping can lead to significant issues and erroneous outcomes when assessing cluster parameters such as the number of member stars, core radius, cluster mass, and overall cluster size, among others. Furthermore, the King model is capable of distinguishing the background level from member stars, which allows for minimal clipping. We only limit the Gaia data to a parallax between 0.03 and 0.9 mas. However, when calculating the average parallax and proper motion, the averages are calculated with errors of 10% of their values. The clipping of the data typically reduces the number of member stars rather than the number of field stars. As an example, in Fig.1, we plot the radial density profile (RDP) of unselected stars in case of the parallax condition $0.2 \le \varpi \ge 0.4$. This figure shows that the clipping data is mostly overdensity of member stars. It is essential and crucial to construct the RDP of the unselected stars in order to determine whether any structure or overdensity remains.

**Fig. 2:**
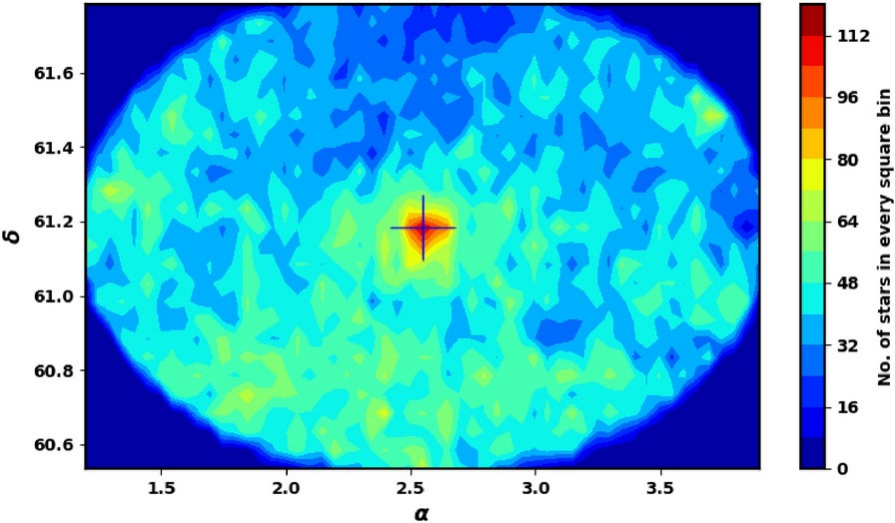
The number surface density of King 13 using the data of Gaia DR3. It’s clear that the cluster is elongated with the average angle of $\theta$ .

**Fig. 3:**
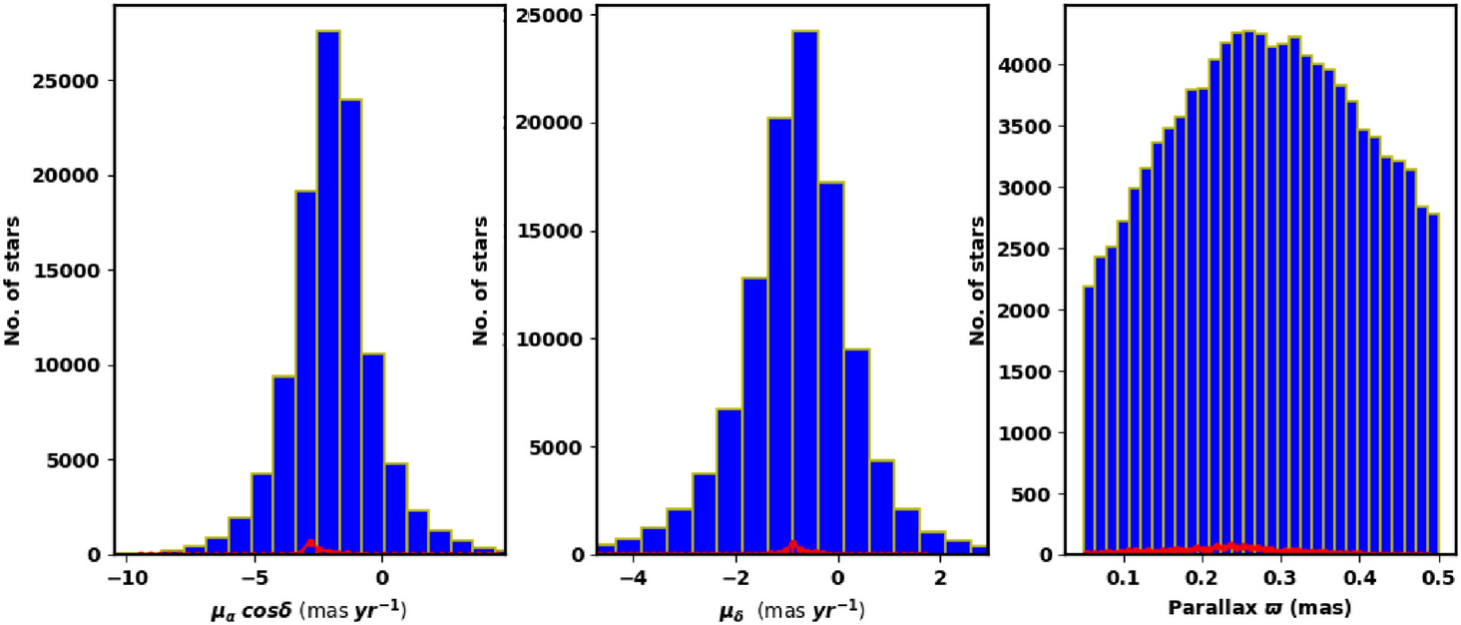
The proper motion in right ascension, declination, and parallax in the field of King 13.

To better constrain the cluster members and determine other astrophysical parameters, we also incorporated infrared data from the 2MASS survey. Fig. 2 shows the surface number density of King 13 derived from Gaia DR3, while Fig. 3 shows histograms of the proper motions ($\mu _{\alpha }\cos \delta$, $\mu _{\delta }$) and parallax ($\varpi$).

### **2MASS data**

The Two Micron All-Sky Survey (2MASS;^36^) utilized two automated 1.3m telescopes, located at Mt. Hopkins, Arizona (USA) and the Cerro Tololo Inter-American Observatory (CTIO) in Chile. Each telescope was equipped with a three-channel camera, featuring a 256 $\times$ 256 array of HgCdTe detectors for each channel. The 2MASS catalog provides photometric measurements in the J (1.25 $\mu$m), H (1.65 $\mu$m), and K$_s$ (2.17 $\mu$m) bands, covering millions of galaxies and nearly half a billion stars. The catalog’s sensitivity reaches magnitudes of 15.8 in J, 15.1 in H, and 14.3 in K$_s$ at a signal-to-noise ratio (S/N) of 10.

## **Cluster structure and radial density profile**

To analyze the cluster structure and construct the radial density profile (RDP), the first step is to accurately determine the cluster’s center. The primary objective is to locate the region with the highest stellar density. To achieve this, we generated a two-dimensional histogram of star counts in right ascension ($\alpha$) and declination ($\delta$) using data from the Gaia DR3 database. By utilizing the histogram2d function from the numpy package, we identified the cell containing the maximum number of stars. This procedure was repeated in Section Proper Motion, Membership Determination, and Cluster Center, focusing exclusively on member stars, with no significant difference observed. To evaluate the extent of the cluster, we constructed the RDP of King 13 by dividing the observed area into concentric rings. The number of stars in each ring, $N_i$, was counted, and the star density was calculated as $n_i = N_i / A_i$, where $A_i$ represents the area of the *i*-th ring ($\pi (R_{i+1}^2 - R_i^2)$). Here, $R_i$ and $R_{i+1}$ correspond to the inner and outer radii of each ring, respectively.

The stars density function $n_{t}(r)$, which represents the total stellar density (including field stars and cluster members), is expressed as:

1 $$\begin{aligned} n_{t}(r) = n_{bg} + n_{c}(r), \end{aligned}$$

where $n_{c}(r)$ is the density of cluster member stars, defined as^37^:

2 $$\begin{aligned} n_{c}(r) = \dfrac{n_{o}}{1+(r/r_{c})^{2}}, \end{aligned}$$

with $r_c$, $n_{bg}$, and $n_o$ representing the core radius, background density, and central density, respectively. The core radius, $r_c$, is the distance from the cluster center at which the stellar density, $n_{c}(r)$, becomes half of the central density $n_{o}$.

An additional parameter, the limiting radius $r_{lim}$, was introduced by^38^. This radius is determined by comparing $n_c(r)$ in equation 2 to the background density threshold, $3 \sigma _{bg}$, defined as:

3 $$\begin{aligned} n_c(r_{lim}) = \dfrac{n_{o}}{1+(r_{lim}/r_{c})^{2}} =3 \sigma _{bg}, \end{aligned}$$

where $\sigma _{bg}$ is the uncertainty in $n_{bg}$. The limiting radius is then calculated as:

4 $$\begin{aligned} r_{lim} = r_c \sqrt{\frac{n_o}{3 \sigma _{bg}} - 1}. \end{aligned}$$

For King 13, $r_{lim}$ is found to be approximately 20.4 $\pm$ 0.87  arcmin. However, this value appears unrealistic due to its dependence on $\sigma _{bg}$, which is influenced by background and foreground star densities and lacks a strong physical basis.

**Fig. 4:**
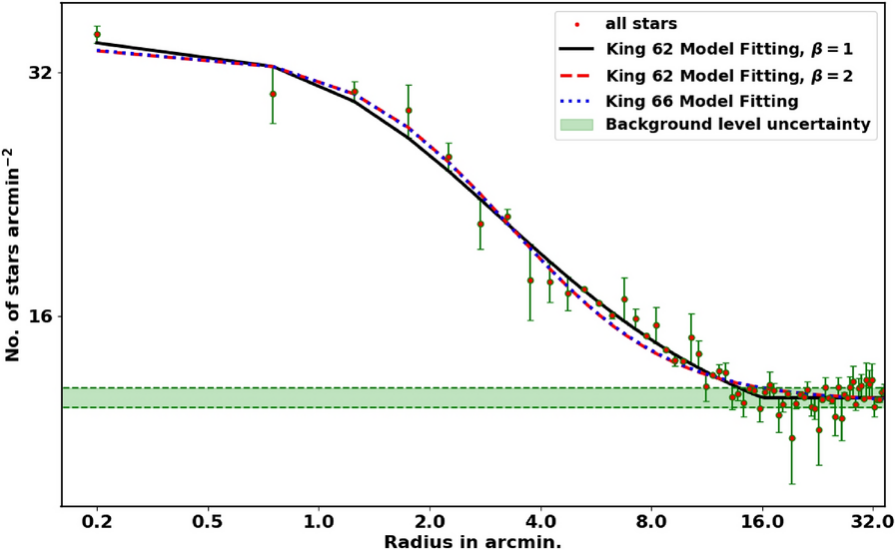
The radial density profile (RDP) of King 13. The solid black and dashed red lines represent the King model fits with $B=1$ and $B=2$, respectively, while the dashed blue line corresponds to the King 66 model. The light green band indicates the background level uncertainty.

Another density model formula, in literature is:

5 $$\begin{aligned} n_t(r) = {\left\{ \begin{array}{ll} n_{bg} + k \left[ \dfrac{1}{\sqrt{1+(r/r_c)^2}} - \dfrac{1}{\sqrt{1+(r_t/r_c)^2}} \right] ^{\beta }, & r \le r_t, \\ n_{bg}, & r > r_t, \end{array}\right. } \end{aligned}$$

and *k* is:

6 $$\begin{aligned} k = n_o \left[ 1 - \dfrac{1}{\sqrt{1+(r_t/r_c)^2}} \right] ^{-\beta } \end{aligned}$$

it is a origin version of Equation 2, and it was introduced by^39^ with $\beta =2$. The tidal radius, $r_t$, is the distance at which the member star density drops to zero. For King 13, we adjusted the power $\beta$ equal to 1 instead of 2, to avoid obtaining unphysically high $r_t$ values. This modified model ($\beta =1$) offers improved accuracy over Equation 2 (see Fig. 4), but this is not a general case and the choice is dictated by the steepness of the density profile of the cluster.

Additionally, the stellar density $n_{c}(r_i)$ in the *i*-th ring can be used to estimate the number of member stars in that ring:

7 $$\begin{aligned} N_{cl, i} = n_{c}(r_i) \cdot A_i, \end{aligned}$$

where the ring radius is defined as:

8 $$\begin{aligned} r_i = \frac{R_{i} + R_{i+1}}{2}. \end{aligned}$$

By summing the cluster density profile up to $r_t$, we estimated the total number of cluster members:

9 $$\begin{aligned} N_{cl} = \sum _{r=0}^{r_t} \left[ N_i - n_{bg} \cdot A_i \right] . \end{aligned}$$

This value serves as a constraint for the membership probability cutoff as discussed in the next section.

The structural parameters of King 13 were determined by fitting the King model to the RDP. The background density, $n_{bg}$, was found to be 13.54$\pm$ 0.03 stars arcmin$^{-2}$ (indicated by the blue dashed line in Fig. 4). The central density, core radius, and tidal radius were determined to be 22.70$\pm$ 0.71 stars arcmin$^{-2}$, 1.60 $\pm$ 0.02 arcmin, and 15.75$\pm$ 4.08 arcmin, respectively (see Table 1). Additionally, the tidal radius $r_t$ and the total number of member stars $N_{cl}$ were estimated as 19.38 $\pm$ 0.94  stars arcmin$^{-2}$ and 1571 $\pm$ 41  stars, respectively.

The uncertainties in the fitted parameters were estimated using the covariance matrix obtained from the curve_fit function in the scipy package (https://scipy.org/).

To quantify the compactness of King 13, we calculated the star density contrast:

10 $$\begin{aligned} \delta _c = 1 + \frac{n_o}{n_{bg}}. \end{aligned}$$

For King 13, $\delta _c$ was found to be 2.68 $\pm$ 0.02 , significantly lower than typical values for compact clusters ($7 \le \delta _c \le 23$) reported by^40^, suggesting that King 13 is a sparse cluster.

Finally, the tidal radius was also estimated using the formula by^41^:

11 $$\begin{aligned} R_t = 1.46 \cdot M_c^{1/3}, \end{aligned}$$

where $M_c$ is the total cluster mass. For King 13, this yielded a tidal radius of 17.95 pc, based on a total mass of 2658.4$\pm$ 61.5  $M_\odot$ (see Section Cluster Mass and Mass Function). This value is near to the tidal radius obtained from the King model fit ($r_t = 18.38$ pc in Equation 5).

Our results are comparable to those of^18^; however, due to our approach to identify the tidal radius and members in each shell, our value of $r_t$ exceeds theirs by approximately 8 pc.

**Table 1:** King model fit parameters.

$n_o$	$n_{bg}$	$r_c$	$r_t$	Model	Ref.
stars/arcmin	stars/arcmin	arcmin	arcmin		
22.70$\pm$ 0.71	13.54$\pm$ 0.03	1.60 $\pm$ 0.02 (1.9 pc)	15.75$\pm$ 4.08 (18.7 pc)	equation 5	this work
36.3 ±4.2	16.±0.3	0.6±0.1	8.5 pc	equation 2	^33^
–	–	3.78 pc	13.9 pc	equation 5	^19^
–	–	1.54 pc	10.59 pc	equation 5	^18^
–	–	2.18 ± 0.27 pc	3.45 ± 1.70 pc	equation 2	^38^

## Proper motion, membership determination, and cluster center

The determination of fundamental parameters for star clusters is often complicated by contamination from field stars. Historically, cluster membership was determined using photometric and kinematic data^42–44^. However, with the advent of astrometric data from the Gaia survey, the accuracy of kinematic methods for membership determination has significantly improved. Proper motion and parallax data are particularly effective in distinguishing field stars from cluster members, as stars in a cluster tend to share similar kinematic properties and distances^45^. In this work, we used Gaia DR3 proper motion and parallax data to differentiate cluster members from nonmembers.

### The membership method: HDBSCAN algorithm

We employed the Unsupervised Photometric Membership Assignment in Stellar Clusters (UPMASK) algorithm, developed by^46^. This is a non-parametric and unsupervised approach that removes the need for prior field star selection. A refined version, available as the *pyUPMASK* Python package (https://github.com/msolpera/pyUPMASK)^47^, extends the original algorithm by incorporating several clustering methods from the scikit-learn library^48^ (https://scikit-learn.org/stable/). This library includes mort than a dozen different clustering methods for unlabeled data, which are all available to use in pyUPMASK, such as KMS, OPTICS, Mini Batch K-means (MBK), Gaussian Mixture Models (GMM) and Hierarchical Density-Based Spatial Clustering of Applications with Noise (HDBSCAN), their refernces are in^47^. This allows for more flexible analysis of unlabeled data.

In this work, we have used HDBSCAN algorithm^49^, and it is implemented in Python by^50^. This HDBSCAN algorithm can be regarded as one of the fastest extant clustering algorithms and it is an advance of both DBSCAN and OPTICS. Notably, DBSCAN operates under the premise that the criteria for clustering, specifically the density requirement, is uniform across the entire dataset. Consequently, DBSCAN may encounter difficulties in effectively identifying clusters that exhibit varying densities. HDBSCAN addresses this limitation by relaxing the uniformity assumption and examining a range of density levels through the development of a different representation of the clustering challenge. Moreover, it predominantly employs a k-means clustering algorithm, a method used for categorizing data by assessing its closeness to designated center points. Furthermore, It is effective at identifying and removing noise in a data set. Then, DBSCAN is one of the most commonly used and cited clustering algorithms.

**Fig. 5:**
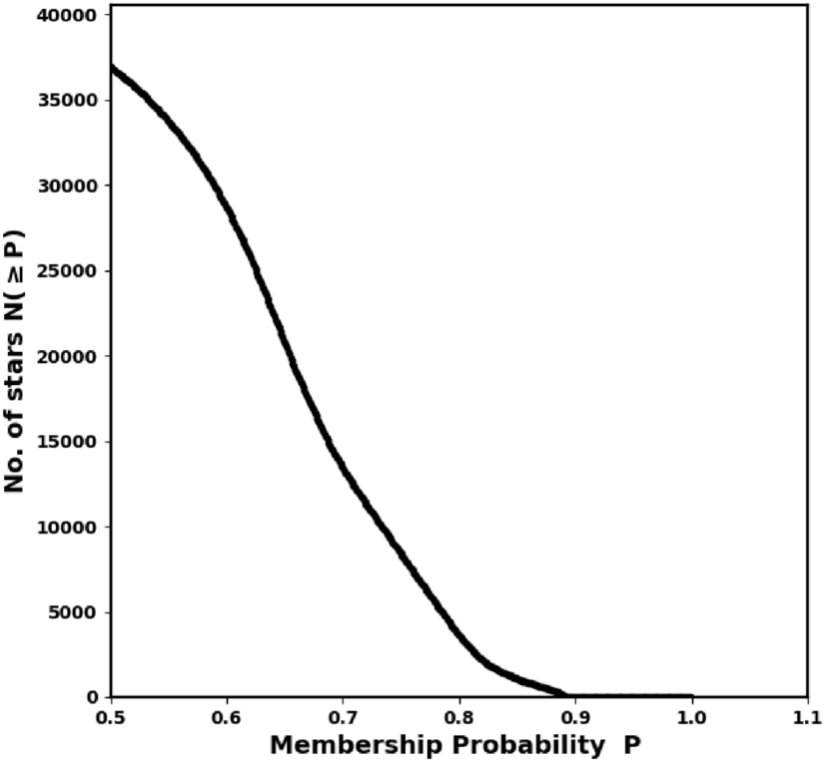
The number of stars as function of membership probability as extracted from *pyUPMask* code.

In this study, we utilized the *pyUPMASK* package with HDBSCAN algorithm to calculate membership probabilities for stars within the cluster. Gaia DR3 data ($\alpha$, $\delta$, $\mu _{\alpha } \cos \delta$, $\mu _{\delta }$ and $\varpi$) for approximately 33,926 stars within a $70^\prime$ radius were used as input. Fig. 5 displays the total number of stars, N($\ge$P), as a function of their membership probability P.

### The probability cut-off value

A membership probability cut-off of 50% is commonly used in membership determination; however, this threshold is not always appropriate. The optimal cut-off depends on the method employed, as well as factors such as the surrounding field density and the distance between the star and the cluster center. Furthermore, the ideal probability cut-off value can vary from cluster to cluster. Therefore, it is crucial to carefully test the choice of probability cut-off, as an incorrect threshold could lead to misclassification of cluster members. Recent studies have used different probability cut-off values. For instance,^27^ used the same method (HDBSCAN) as we did, with a probability cut-off of 50%. On the other hand,^51,52^ applied UPMASK with a probability cut-off of P> 70% and the GMM model with P > 80%, respectively. Thus, the choice of the probability cut-off value remains a subject of ongoing debate.

**Fig. 6:**
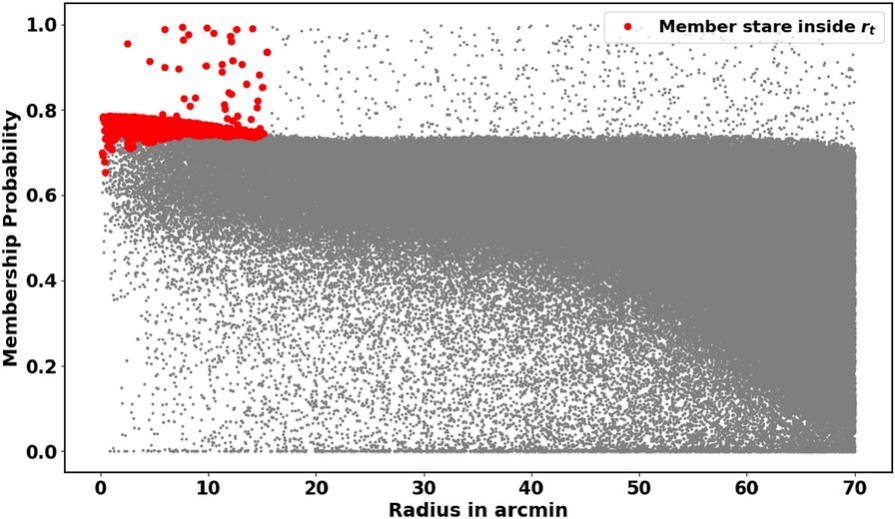
Our approach entails examining the Probability $P_{i}$ at each range as a function of the radius $r_i$. For detecting the tidal tail, we extend the probability outside $r_t$.

**Fig. 7:**
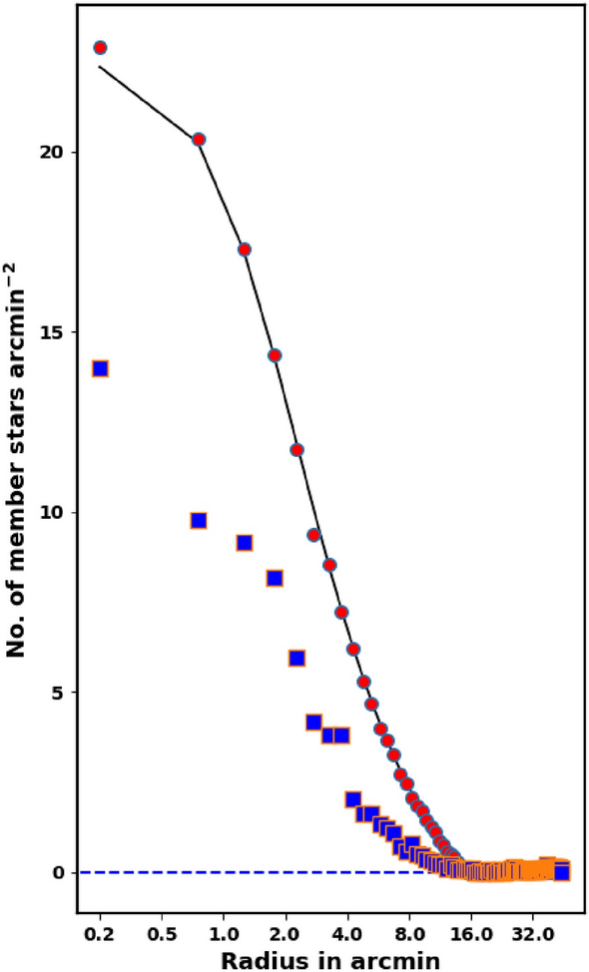
The stellar density profile of member stars. The red dots represent the density of probable member stars from this work, while the solid line corresponds to the fitted King model density profile of the cluster, based on equation 5. Additionally, the member stars identified by^19^ are shown as blue squares.

In general, there are two types of probability cut-off values: (i) a single cut-off value applied to the entire cluster, and (ii) a cut-off value applied at each radial distance, $r_i$. In this study, we adopt the latter approach by using a radius-dependent cut-off value (see Fig. 6). The fitted King profile model also plays a significant role in this process. This method is crucial for obtaining a more accurate analysis of the cluster’s morphology and for investigating the structure of its tidal tail. The approach is mathematically:

12 $$\begin{aligned} Nm_{i}(P\ge P_i) \;\approx \; n_{c}(r_i) \; A_i \end{aligned}$$

where $P_i$ is the probability in the *i*-th ring, giving the number of member stars as $Nm_{i}$, which should match the number of stars from the King model, $n_{c}(r_i) \; A_i$, as shown in Fig. 7. We can identify these member stars in Python using the following index:

13 $$\begin{aligned} ind_{memb,i} = \left( P \ge P_i \right) \end{aligned}$$

**Fig. 8:**
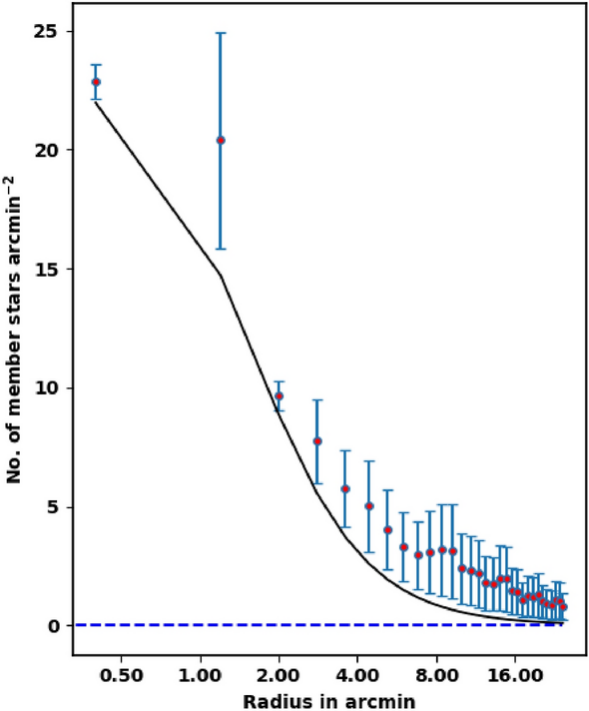
The cluster stellar density at probability cut-off value 70%. The members at this probability cut-off are much over King model function which are overestimated.

The King density profile is a valuable tool for verifying the accuracy of both the membership separation method and the total number of cluster members. Conversely, an incorrect membership separation method or an improper probability cut-off could lead to an overestimation or underestimation of the number of member stars. For example, using a cut-off of 70% would result in an overestimation of the member stars, as illustrated in Fig. 8. In comparison with previously reported membership estimates by^19^ (see Fig. 7), one can note that they underestimated the number of King 13 members.

## The color magnitude diagrams and cluster age

Color-magnitude diagrams (CMDs) for OCs employ empirical isochrones to compare with theoretical models of stellar evolution^53,54^. CMDs serve as effective tools for estimating key parameters such as distance, age, and metallicity of a cluster. Additionally, by comparing observed CMDs with theoretical isochrones, valuable insights into the masses of stars within the cluster can be obtained. The theoretical isochrones used in this study were downloaded from the CMD 3.7 website (http://stev.oapd.inaf.it/cgi-bin/cmd), utilizing PARSEC version 1.25s^55^.

### **Extinction**

A precise interstellar dust extinction law is critically important for interpreting observations. Extinction coefficients for each passband depend on the source’s spectral energy distribution, interstellar matter, and the extinction itself. Both the color excess ratio (CER), $E(\lambda -\lambda _1)/E(\lambda _2-\lambda _1)$, and the relative extinction, $A_\lambda /A_{\lambda _1}$, are key indicators of the extinction law.

We follow^56^ and use the method presented in^57^, we compute the extinction coefficients in the Gaia and 2MASS bands using the relation $A_{\lambda } = a A_V$. For example:

$$A_G/A_V = 0.789, \quad A_{BP}/A_V = 1.008, \quad A_{RP}/A_V = 0.589$$

For 2MASS bands:

$$A_J/A_V = 0.243, \quad A_{K_s}/A_V = 0.078, \quad A_H/A_V = 0.131$$

For the 2MASS observations, we adopt the extinction law values of^58^ and^59^:

$$A_U/A_V = 1.558, \quad A_B/A_V = 1.326, \quad A_R/A_V = 0.81, \quad A_I/A_V = 0.56$$

Using these values, the relation between extinction and color excess can be expressed as follows:

$$\begin{aligned} A_J&= 1.473 \times E(J-K_s)&\\ A_G&= 1.88 \times E(G_{BP} - G_{RP})&\end{aligned}$$

and then,

14 $$\begin{aligned} E(G_{BP} - G_{RP}) = 2.6\times E(J-K_s) \end{aligned}$$

From isochrone fitting, we estimate the color excess and, consequently, the extinction. Using the following equation, the intrinsic distance modulus $(m-M)_0$ can be calculated:

15 $$\begin{aligned} \left( m-M \right) _{\text {obs}} = \left( m-M \right) _{0} + A_{\lambda } \end{aligned}$$

where *m* is the apparent absorbed magnitude, *M* is the absolute magnitude, and $A_{\lambda }$ is the extinction in the $\lambda$ band.

### **The CMD Of Gaia DR3 data**

**Fig. 9:**
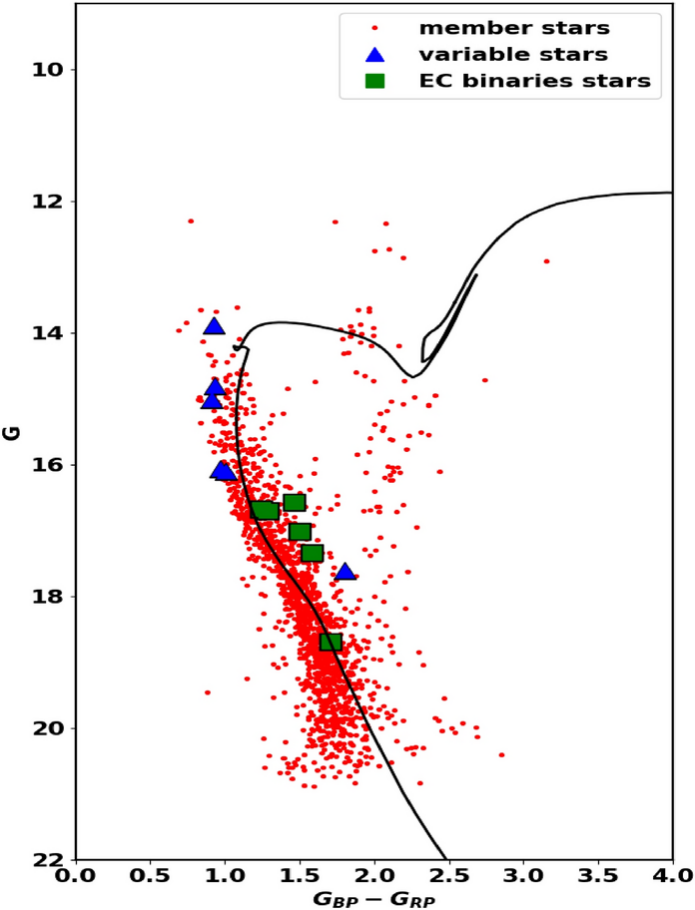
The color-magnitude diagram (CMD) for all the member stars of King 13 with probability as a function of different radii, based on the Gaia DR3 photometric bands (G, $G_{BP}$, and $G_{RP}$). Blue triangles represent stars flagged as variable in Gaia archive while the green squares are for the eclipsing binary stars^60^.

Using the photometric data from Gaia DR3 for stars in King 13, the color-magnitude diagram (CMD) is presented in Fig. 9. The CMD is fitted with theoretical isochrones from^53^.

We determined the observed distance modulus and the color excess, E(G$_{BP}$ - G$_{RP}$), to be 14.85 mag and 1.17 $\pm$ 0.07 mag, respectively. The true distance modulus $\left( m - M \right) _{o}$ and the extinction in the G band ($A_G$) were calculated using the equation provided in Section Extinction, yielding values of 13.11$\pm$ 1.03 and 2.21, respectively. These results correspond to an isochrone-based distance ($d_{iso}$) of 4187$\pm$ 262 pc. Furthermore, the fitted isochrone indicates a cluster age of approximately 310$\pm$ 28 Myr, with a metallicity of Z = 0.015, see Table 2. Fig. 9 illustrates the CMD for King 13, utilizing the broad photometric bands (G, G$_{BP}$, and G$_{RP}$) from the Gaia DR3 dataset and considering all the cluster’s members counted at different radii. Some of the members appear to be parallel to the CMD to the right and could be long period variables not yet identified by Gaia.

#### Variability and binaries

Gaia DR3 introduces significantly improved data products compared to the earlier EDR3 release. This update includes the first Gaia catalog of eclipsing binary candidates, comprising 2,184,477 sources with brightness levels ranging from a few magnitudes up to 20 magnitudes in the Gaia G-band, and covering the entire celestial sphere^60^. By cross-matching our member stars with this catalog, we identified 6 members as eclipsing binaries, indicated by green squares in Fig. 9. Additionally, these stars are flagged as variable stars among the 12 variable stars in the Gaia Archives. Despite the importance of studying variable stars, including binaries (e.g.,^61–64^), a detailed analysis of these systems is beyond the scope of this paper and will be addressed in future studies.

### **The CMD of 2MASS data**

**Fig. 10:**
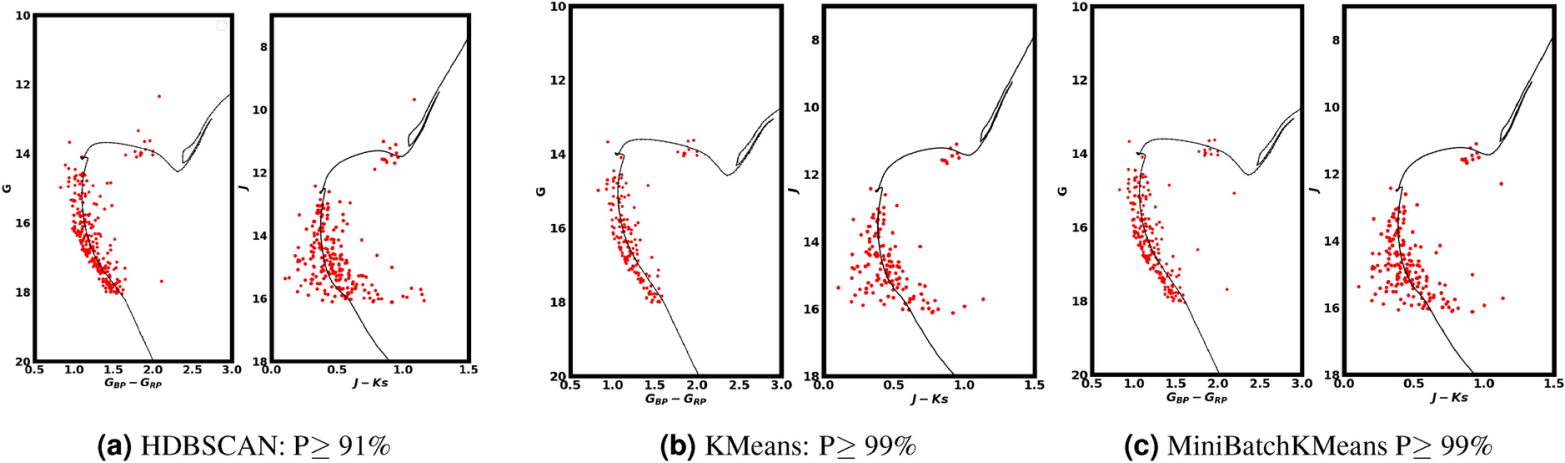
Gaia and 2MASS CMDs of the 430 stars (most probable members) identified using different methods, including HDBSCAN, KMeans, and MiniBatchKMeans. Black triangles and blue dots overplotted on the Gaia CMD represent results from previous studies conducted by^66,67^.

Using the intersect1d function from the Python **NumPy** package (https://numpy.org/)^65^, we matched the member stars identified in Gaia DR3 with the 2MASS dataset. The resulting color-magnitude diagram (CMD) of the matched 2MASS data was used to validate the effectiveness of the membership separation method. We then plotted the CMDs, as shown in Fig. 10. By performing isochrone fitting, we derived the color excess value, $E(J-K_s)$ ( 0.44 $\pm$ 0.03 mag). Using the equations outlined in Section Extinction, the extinction value $A_J$ was calculated to be $0.63 \pm 0.071$. Finally, the distance modulus was determined to be 13.08$\pm$ 0.12 , corresponding to a physical distance of 4140 $\pm$ 236 pc, see Table 2. This result is in excellent agreement with the distance derived from Gaia parallax and photometry, for further validating our analysis. It is important to note that Fig. 10 displays the CMDs for both 2MASS and Gaia data, focusing on the most probable members identified using different approaches, including HDBSCAN, KMeans, and MiniBatchKMeans. The total number of high-probability members identified through these methods is 395.

**Table 2:** Photometric parameters of King 13.

age	$E(G_{BP}-G_{RP})$	$A_{G}$	$(m-M)_{Gaia}$	$E(J-K_s)$	$A_J$	$(m-M)_{2MASS}$	Ref.
Myr	mag	mag	mag	mag	mag	mag	
310$\pm$ 28	1.17 $\pm$ 0.07	2.21	13.11$\pm$ 1.03	0.44 $\pm$ 0.03	0.63	13.08$\pm$ 0.12	This work
510±60	0.71$\pm 0.26$	1.44	-	0.48±0.06	-	14.8 ± 0.2	^33^
794.	–	-	-	0.28	–	12.81	^38^
395	–	-	-	0.39	–	12.72	^68^
313	–	-	-	-	–	-	^43^
310	–	-	-	-	–	12.5 ± 0.2 (BV data)	^30^

### **Luminosity function**

**Fig. 11:**
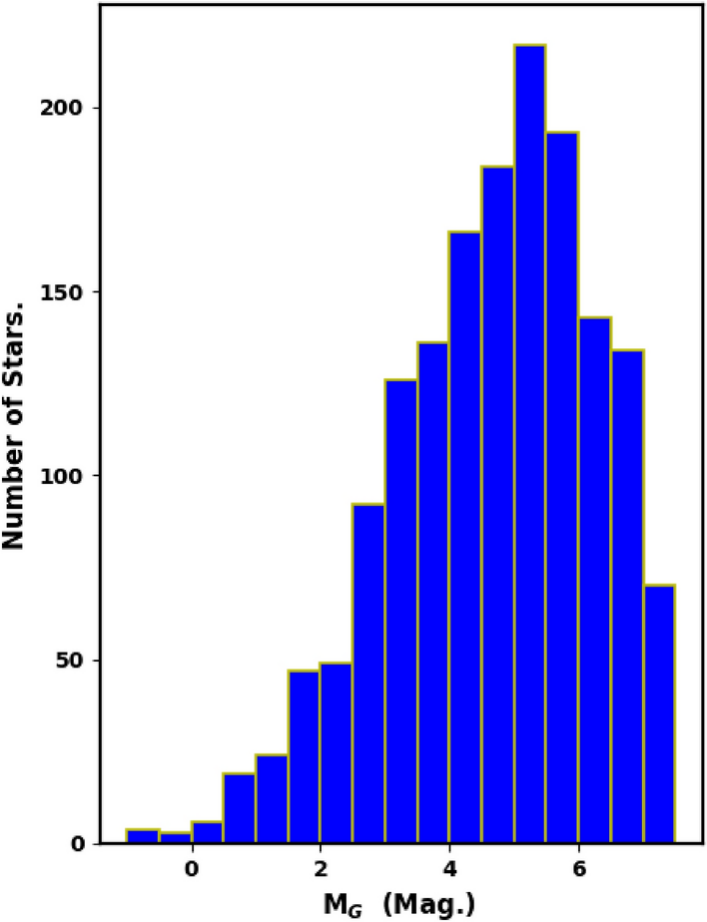
The luminosity function (LF) of King 13, with an interval of 0.5 mag.

The luminosity and mass functions (LF and MF) are closely tied to the cluster’s membership. To effectively eliminate contamination from field stars in King 13’s main sequence, we utilized the *pyUPMASK* Python package to select probable cluster members. After this, the photometric data were used to derive the luminosity function (LF) before proceeding to estimate the mass function (MF). For the LF, the apparent G magnitudes of the member stars were converted into absolute magnitudes, and histograms were created to display the LF of King 13 (Fig. 11).

### **Cluster mass and mass function**

The individual stellar masses, alongside the total cluster mass, play a crucial role in understanding a cluster’s properties. After performing the isochrone fitting, we obtained the absolute magnitude, M$_G$, and the intrinsic color, $G_{BP}-G_{RP}$. Using simple polynomial fitting to calculate stellar masses can produce erroneous results due to its limitations. Thus, we employed an interpolation routine with two independent variables. Specifically, we used the *SmoothBivariateSpline* function from the Python Scipy (https://scipy.org/) package^69^, which allows interpolation with two variables, as stellar mass is dependent on both magnitude and color.

We used $M_G$ and $(G_{BP}-G_{RP})_o$ as independent variables from the best-fit isochrone to interpolate individual stellar masses. This approach enabled us to accurately determine the mass of each cluster member, yielding a total cluster mass of M$_c$ = 2658.4$\pm$ 61.5   $M_\odot$. Additionally, we computed the cluster’s mass profile, as shown in Fig. 19, and the derived half-mass radius, $R_h$= 5.4 $\pm$ 0.85 arcmin, where half of the total mass is enclosed it and this value will be used in equation 19.

The mass function (MF) describes the distribution of stellar masses within a cluster per unit volume during significant star formation. A key debate in astrophysics studies has been whether the initial mass function (IMF) is universal or shaped by the conditions and environments present during star formation. This remains an active area for research, as noted in studies by^70–72^. Additionally, investigating mass segregation in open clusters improves our understanding of the distribution of low-and high-mass stars within the cluster.

**Fig. 12:**
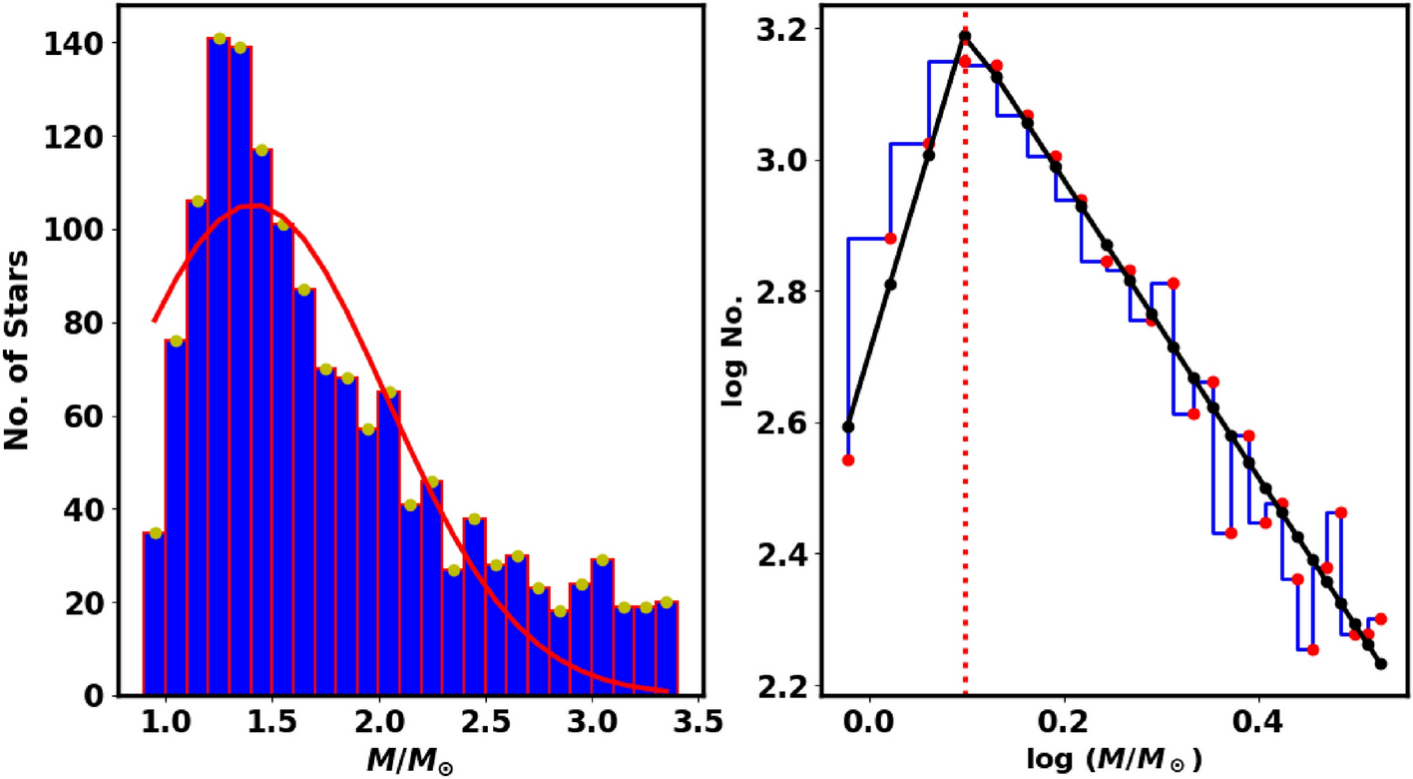
Left panel: Mass histogram with a red line indicating the Gaussian fit, which has a mean of approximately 1.27 $M_{\odot }$ and a standard deviation $\sigma _{M} = 0.35$. Right panel: Mass function (MF) of King 13, where the black solid lines represent two power-law fits with exponents $\alpha _1 =$ -3.7$\pm$0.4 , $\alpha _2$ = 2.3 $\pm$0.15 and a turn off point mass $(M_{\textrm{cr}}$ = 1.25 $\pm$ 0.2 (see Equation 16). The dashed red line marks the critical mass value.

In this work, the MF is mathematically expressed through step function with two parts of power law, as shown by^73^, in contrast to the single power-law equation proposed by^74^. This dual representation provides greater insight into the mass distribution of stars during the early stages of cluster formation, highlighting the complexities of stellar dynamics. It can be represented as follows:

16 $$\begin{aligned} f(M) = \frac{dN}{dM} =\; \Bigg \{ \begin{array}{lcc} K_1 \;\times M^{-\alpha _1} & , & \text {if } M \le M_{cr}\\ K_2 \; \times M^{-\alpha _2} & , & \text {if } M > M_{cr} \end{array} \end{aligned}$$

where dN/dM denotes the number of stars within the mass range *M* to $M + dM$. The $\alpha _1$ and $\alpha _2$ represent the low mass slope and the high mass slope of the mass function, while $M_{cr}$ is the critical mass where the slope changes value and sign.  The fitting is done by curve_fit function in the Scipy python package and $K_1$, $K_2$, $\alpha _1$, $\alpha _2$ and $M_{cr}$ are free parameters, within the mass range of 0.9 to 3.5 $M{\odot }$. The constant $K_1$and $K_2$ must satisfy the condition $f(M^{-}_{cr}) = f(M^{+}_{cr})$ .  For King 13, we have determined that $\alpha _1$, $\alpha _2$ and $M_{cr}$ are -3.7$\pm$0.4 , 2.3 $\pm$0.15 and 1.25 $\pm$ 0.2 $\; M_{\odot }$ (see Fig.  12). The high mass slope $\alpha _2$ value is near to Salpeter value (^74^). Moreover, this $M_{cr}$ value is corresponding to $G \approx 19.37$ Mag.

**Fig. 13:**
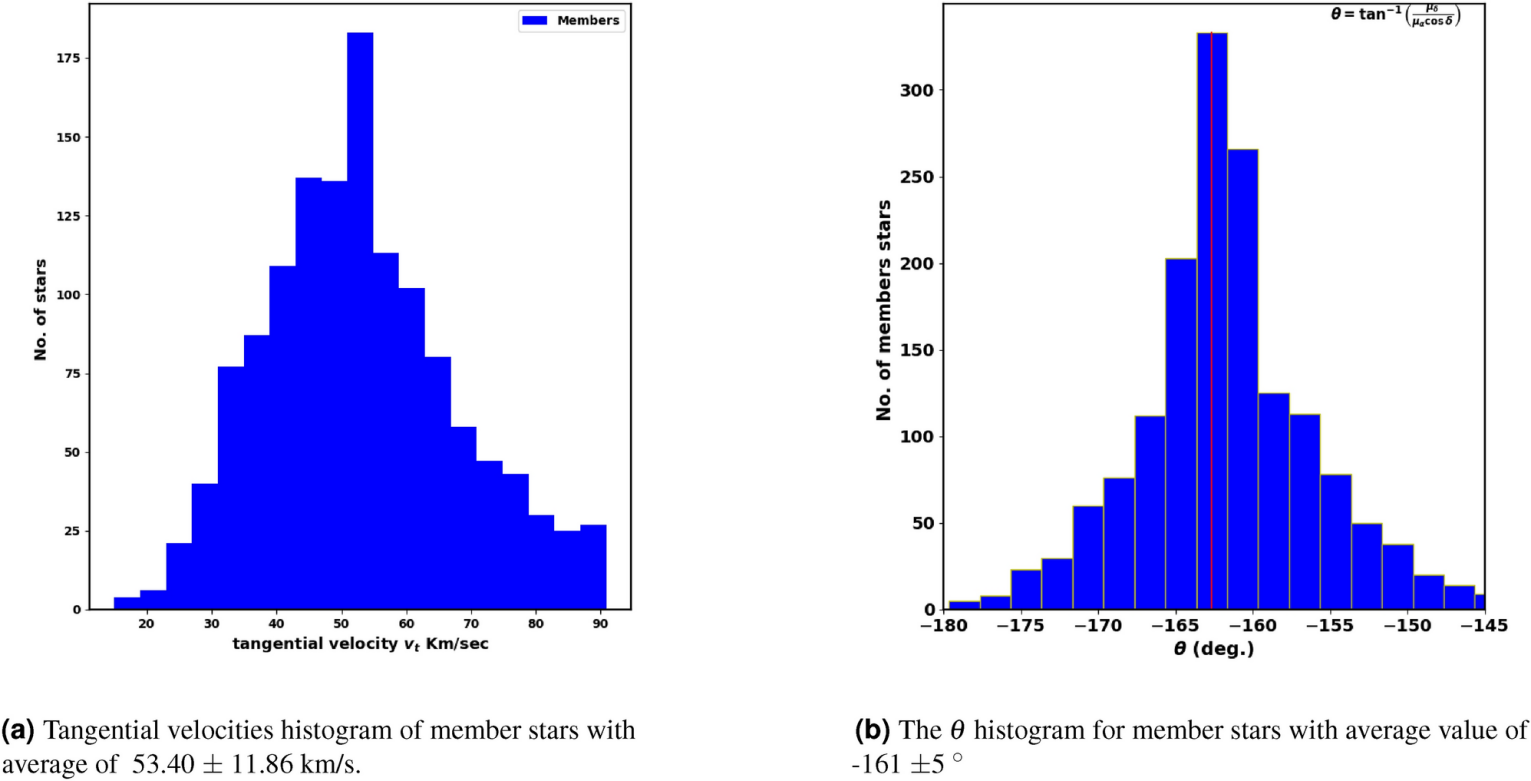
Tangential velocities histogram and their directions.

**Fig. 14:**
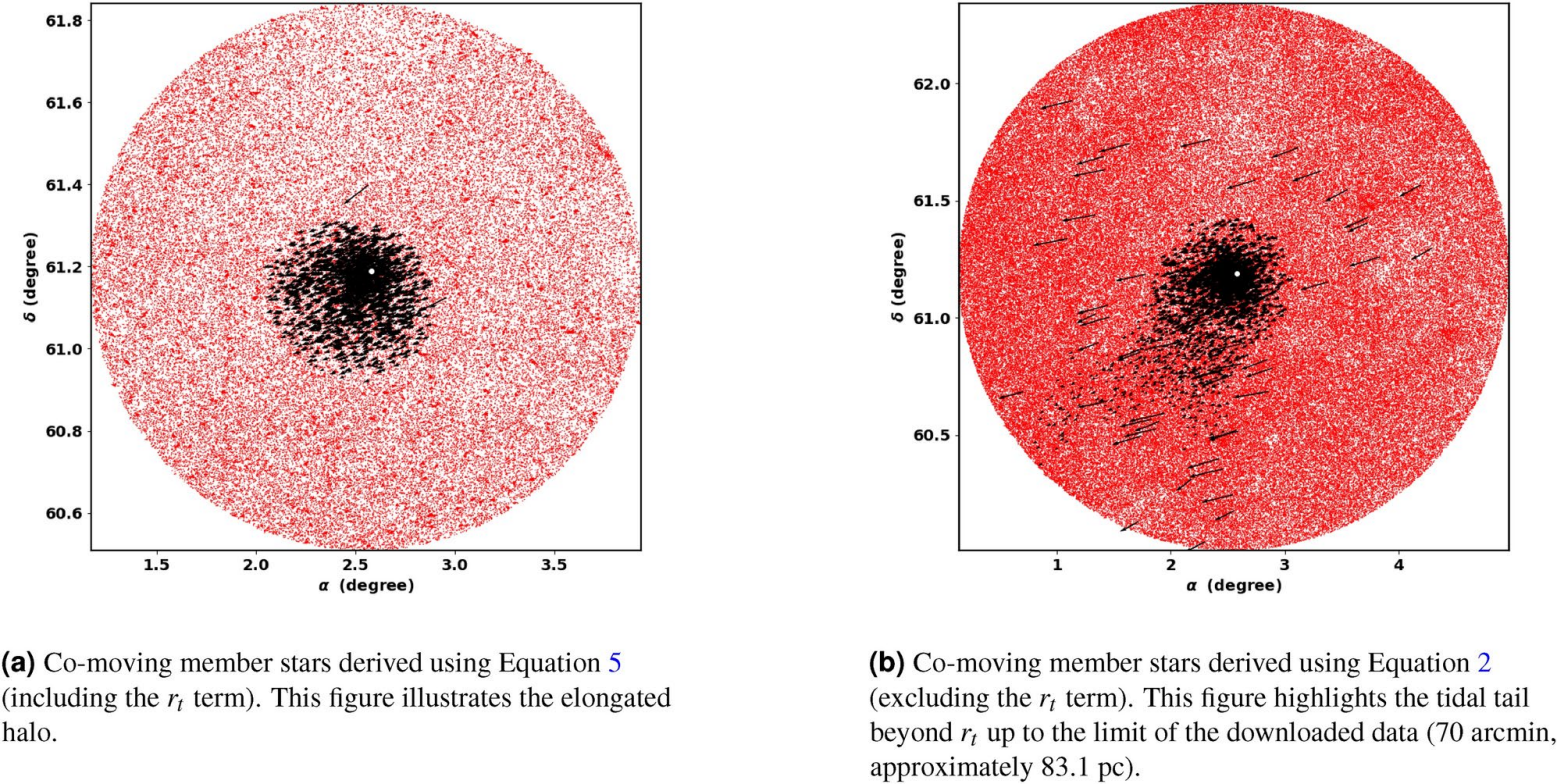
The co-moving stars of King 13 from Gaia DR3.

**Table 3:** Astrometric parameters of King 13, including the cluster center’s coordinates, proper motion, parallax, and the total number of members.

$\alpha$	$\delta$	$\mu _{\alpha }cos\delta$	$\mu _{\delta }$	${\varpi }$	N	Ref.
deg.	deg.	mas yr$^{-1}$	mas yr$^{-1}$	mas	Stars	
2.56 $\pm$ 0.12 $^\circ$( 00:9:43 )	61.17 $\pm$ 0.05 $^\circ$ ( 61:11:59 )	-2.64 $\pm$ 0.36	-0.89 $\pm$ 0.25	0.245 $\pm$ 0.05	1571 $\pm$ 41	This work
–	–	-2.80 ± 0.2	-0.90 ±0.10	0.26±0.01	172	^33^
2.56	+61.18	-2.76	-0.83	0.228	279	^75^
2.55	+61.18	-2.75	-0.84	0.231	288	^3,66^

## Cluster kinematics and dynamics

Open clusters are excellent tracers of the evolution of the Galactic disc. Thanks to Gaia DR3, their kinematics can now be investigated with unprecedented precision and accuracy. To accurately determine the cluster parameters, we averaged the values of member stars with a membership probability greater than 95% within a 5 arcmin radius. The center of the cluster is located at 2.56 $\pm$ 0.12 $^\circ$( 00:9:43 ) and 61.17 $\pm$ 0.05 $^\circ$ ( 61:11:59 ) , which corresponds to the Galactic coordinates l= 117.98 $\pm$ 0.06 $^\circ$ and b= -1.31 $\pm$ 0.05 $^\circ$ . Additionally, the proper motion components are $\mu _{\alpha } \cos \delta$ = -2.64 $\pm$ 0.36 mas yr$^{-1}$ and $\mu _{\delta }$ = -0.89 $\pm$ 0.25 mas yr$^{-1}$. The average parallax ($\varpi$) is found to be 0.245 $\pm$ 0.05 mas. The corresponding distance to the cluster, calculated as $d_{\varpi } (\text {pc}) \approx 1000 / \varpi (\text {mas})$, is 4082 $\pm$ 231 kpc. This value is consistent with the results obtained from photometric data, within the associated uncertainties. The detailed results are presented in Table 3. Table 3 also presents a comparison between our results and previously published values, showing good agreement overall. The main discrepancy lies in the number of cluster members since we applied probability as a function of radius. However, our results for the most likely members (395 stars) are somewhat similar to those of previous studies.

It is well-known that the stars in the cluster move at almost the same speeds. Consequently, the tangential velocities of open clusters, obtained from absolute proper motions ( $\mu = \sqrt{ (\mu _{\alpha }\cos \delta )^2 + (\mu _{\delta })^2}$ ) and parallaxes ($\varpi$), enable the identification of the type of orbit the cluster follows. This contributes significantly to studies of cluster origins and destruction processes. The tangential velocity in km/s is given by:

17 $$\begin{aligned} v_t = 4.74 \; \mu \; d \; ; \hspace{1cm} d=\dfrac{1000}{\varpi } \end{aligned}$$

where the constant 4.74 comes from the unit conversion:

$$\begin{aligned} \dfrac{(4.84 \times 10^{-6} \; \text {rad}) \; (3.086 \times 10^{13} \; \text {km})}{(3.154 \times 10^{7} \; \text {s})} \approx 4.74 \end{aligned}$$

Here, *d*, $\mu$, and $\varpi$ are the distance (pc), proper motion (arcseconds yr$^{-1}$), and parallax (arcseconds), respectively. Fig. 13a shows a histogram of tangential velocity $v_t$ with an average value of 53.40 $\pm$ 11.86 km/s, following a nearly Gaussian distribution.

Another key parameter is the angle $\theta$, which indicates the direction of the cluster’s motion in the $\mu _{\alpha } \cos \delta$ and $\mu _{\delta }$ space, as described by this formula:-

18 $$\begin{aligned} \varvec{\theta }= \tan ^{-1}\left( \frac{\mu _{\delta }}{\mu _{\alpha } \cos \delta } \right) \end{aligned}$$

Cluster member stars generally move in nearly the same direction through space. Then $\theta$ is a crucial parameter for testing the membership probability methods. Fig. 13b presents a histogram of $\theta$ for member stars, with an average angle of -161 $\pm$5 $^\circ$ , providing a clearer view compared to Fig. 14. Additionally, the dispersion in the $\theta$ histogram could reflect the cluster’s age and its degree of gravitational binding.

Moreover, utilizing the cluster’s data from Gaia DR3, which contains 46 stars, we calculated the radial velocity of the cluster to have a mean value of approximately -59.7$\pm$ 8.7 km/s. This result is in good agreement with^76^, who estimated it to be -60.35 km/s. Therefore, the cluster space velocity ($v_{space} = \sqrt{v_r^2 + v_t^2}$) is about 87 $\pm$ 13 km/s, making an angle of -47.5$\pm$ 9.2$^{\circ }$ with tangential velocity direction. Then we can get the orbital parameters of the studied cluster, see next subsection Cluster Orbit.

### Cluster orbit

Open clusters (OCs) are excellent tracers of the evolution of the Galactic disc. Thanks to **Gaia DR3**, their kinematics can be investigated with unprecedented precision, particularly for proper motion and parallax ($\mu _{\alpha } \cos \delta$, $\mu _{\delta }$, and $\varpi$). Additionally, Gaia DR3 provides radial velocities (RV) for millions of relatively bright, late-type stars^77^, collected by the Radial Velocity Spectrometer (RVS) instrument^78^. The combination of parallax, proper motion, and RV provides valuable phase-space information. For example,^79^ demonstrated the great potential of Gaia data for studying the kinematics of the Galactic disc and open clusters, revealing the richness of phase-space substructures. Open clusters trace the formation and evolution of our Galaxy. Their ages cover the entire lifespan of the Galactic disc, spanning the young to old thin-disc components. Their spatial distribution and motion help to better understand the gravitational potential and the perturbations acting on the structure and dynamics of the Galaxy. The orbital motions of open clusters are essential not only for understanding their dynamical evolution in the Galaxy but also for investigating the dynamics of the Galaxy itself.

To compute a cluster’s orbit, we must first adopt a model for the Galactic potential. This potential must accurately reproduce the observed mass density of the Galaxy. For this purpose, we performed backward orbital integration of King 13 using the **“MWPotential2014”** model, the default Galactic potential in the *galpy* package^80^. This model consists of three components: **(1)** the Galactic disk potential, defined by the Miyamoto-Nagai expression^81^, **(2)** the bulge component, described by a spherical power-law potential^80^, and **(3)** the dark matter halo potential, given by the Navarro-Frenk-White profile^82^. The Sun’s galactocentric radius, orbital velocity, and *z*-coordinate were taken as $R_{GC}=8$ kpc, $V_{rot}=220$ km/s, and $z=20.8$ pc^80^.

**Fig. 15:**
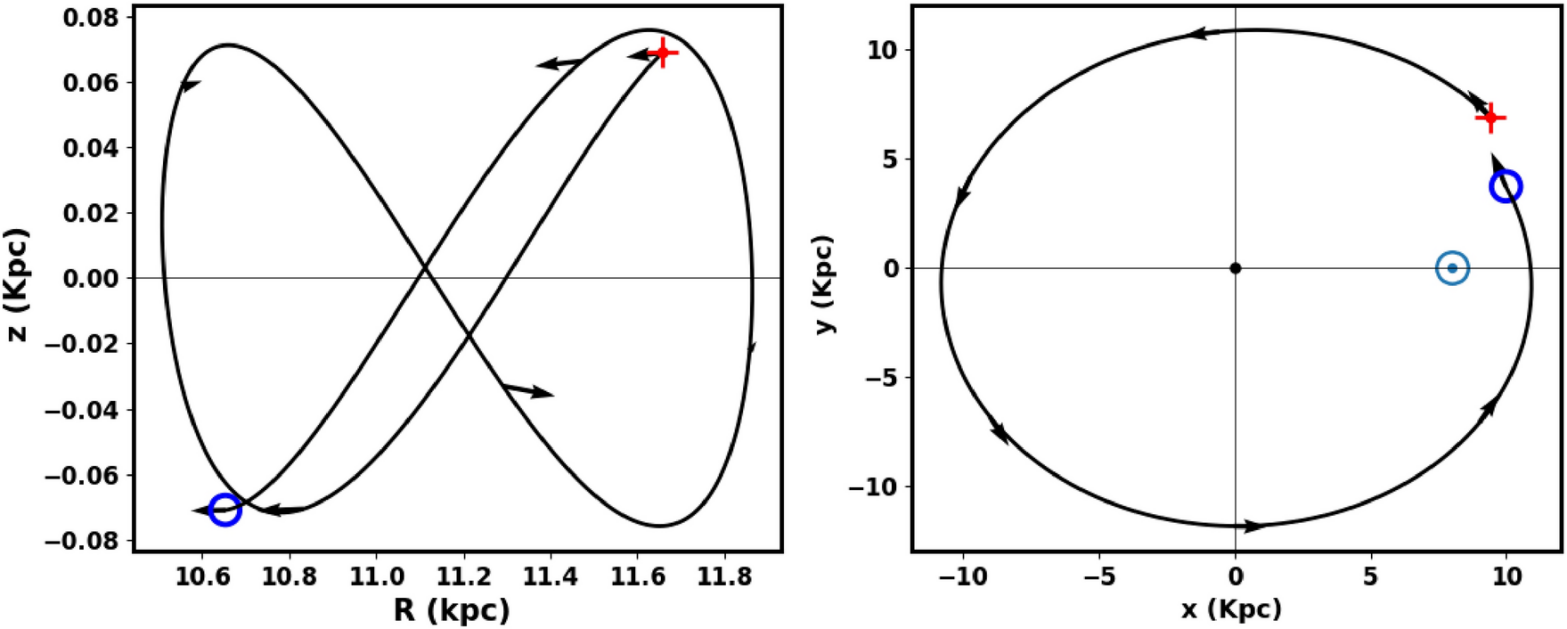
The cluster orbit in 2D artesian Galactocentric coordinates: The red cross is the birth place and the open blue circle is the currant place. The $\odot$ represents the sun position. The cluster moves toward **Galactic center direction, parallel to Galactic plane, the arrows display the direction of motion.**

**Fig. 16:**
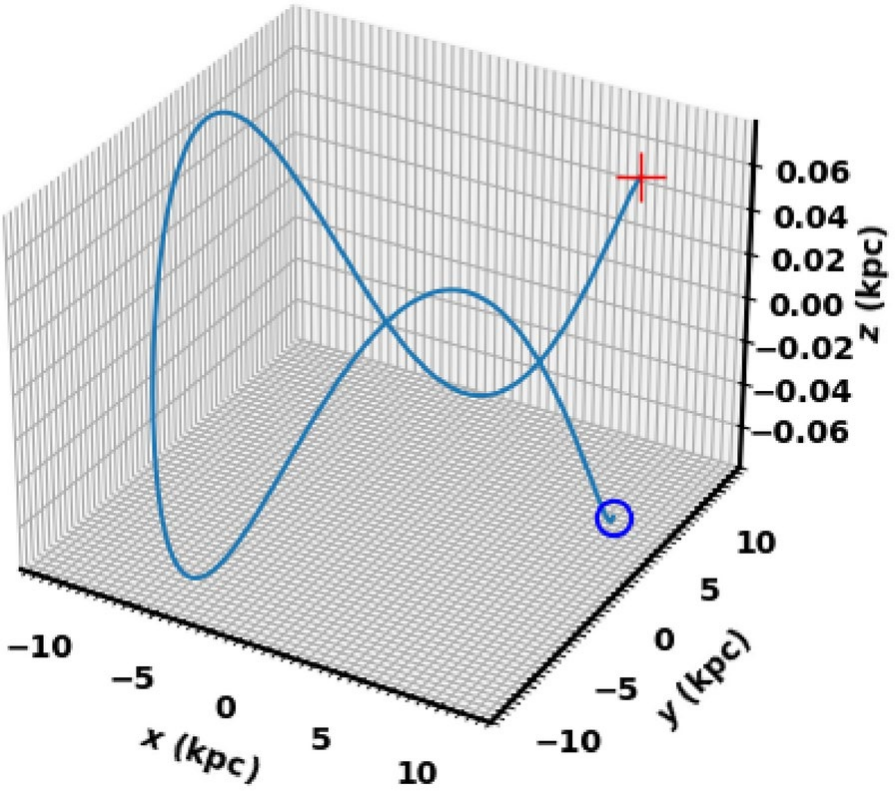
The cluster orbit in 3D Cartesian Galactocentric coordinate system. The red cross is the birthplace.

For input, we used the cluster parameters presented: proper motions ($\mu _{\alpha } \cos \delta$, $\mu _{\delta }$), distance from the Sun, equatorial coordinates ($\alpha$, $\delta$), and radial velocity, which was calculated as an average from the Gaia DR3 data for member stars. Figures 15, 16 show the integrated orbit of King 13 in the Cartesian Galactocentric coordinate system, backward in time according to the cluster age determined in this study. The red cross indicates the birthplace of the cluster. According to the *z*-coordinate, the cluster oscillates about every 321. million years, rising above the plane of the disk up to a maximum height of 76.60 pc, i.e. the cluster is located at the very thin-disk component of the Galaxy. The apocenter $R_{apo}$ and the pericenter $R_{peri}$ are found to be 11.86 and 10.51 Kpc, respectively, which correspond to the eccentricity of the orbit ( $e=( R_{apo}-R_{peri})/(R_{apo}+R_{peri})$) 0.06 and the orbital period $T_r$ = 0.24 Gy. The current coordinates X,Y and Z are 9.97, 3.72 and -0.07 kpc , respectively and the velocities ($v_x$, $v_y$ and $v_z$ ) are -90.5 , 198.9 and -90.5 km/s. Moreover, the space velocity components U, V and W are 79.43 , -33.3 and -7.2 km/s, respectively. The results and comparison with the others are shown in Table 4.

**Table 4:** The orbital parameters of King 13 including, radial velocity, apocenter, pericenter, eccentricity, distance from the Galactic center, orbital period, velocities at the Cartesian coordinates and space velocity components, respectively

Radial Velocity	$R_{apo}$	$R_{peri}$	e	$R_{GC}$	$T_r$	$v_x$	$v_y$	$v_z$	U	V	W	Ref.
km/s	kpc	10.64	-	kpc	Gyr	km/s	km/s	km/s	km/s	km/s	km/s	
-59.7$\pm$ 8.7	11.86	10.51	0.06	10.64	0.24	-90.5	198.9	-0.11	79.43	-33.3	-7.2	this work
-60.35	10.78	9.91	0.04	10.35	-	79.50	220.13	2.16	-	-	-	^76^
-60.35	-	-	-	-	-	-	-	1.49	74.20	-29.08	-5.76	^83^

### The tidal tail

A notable characteristic of open clusters (OCs) is the presence of tidal tails and elongated structure. King 13 is an exceptional cluster because it exhibits both a tidal tail and an elongated halo. We first identified the elongated halo structure using the combined King model^39^ (see equation 5) in conjunction with membership determination by pyUPMASK, as discussed in Section The Probability Cut-off Value. This structure, shown in Fig. 14a, guided our search for the tidal tail. However, the King model in equation 5 is limited to a radius $r_t$, so we extended our investigation beyond it, up to the limit of the downloaded data (70 arcmin), see Fig. 6. As expected, we detected the tidal tail, as shown in Fig. 14b, though its exact extent remains unclear. Recognizing the direction of tidal movement is essential for grasping the physical phenomena that contribute to its behavior.

**Fig. 17:**
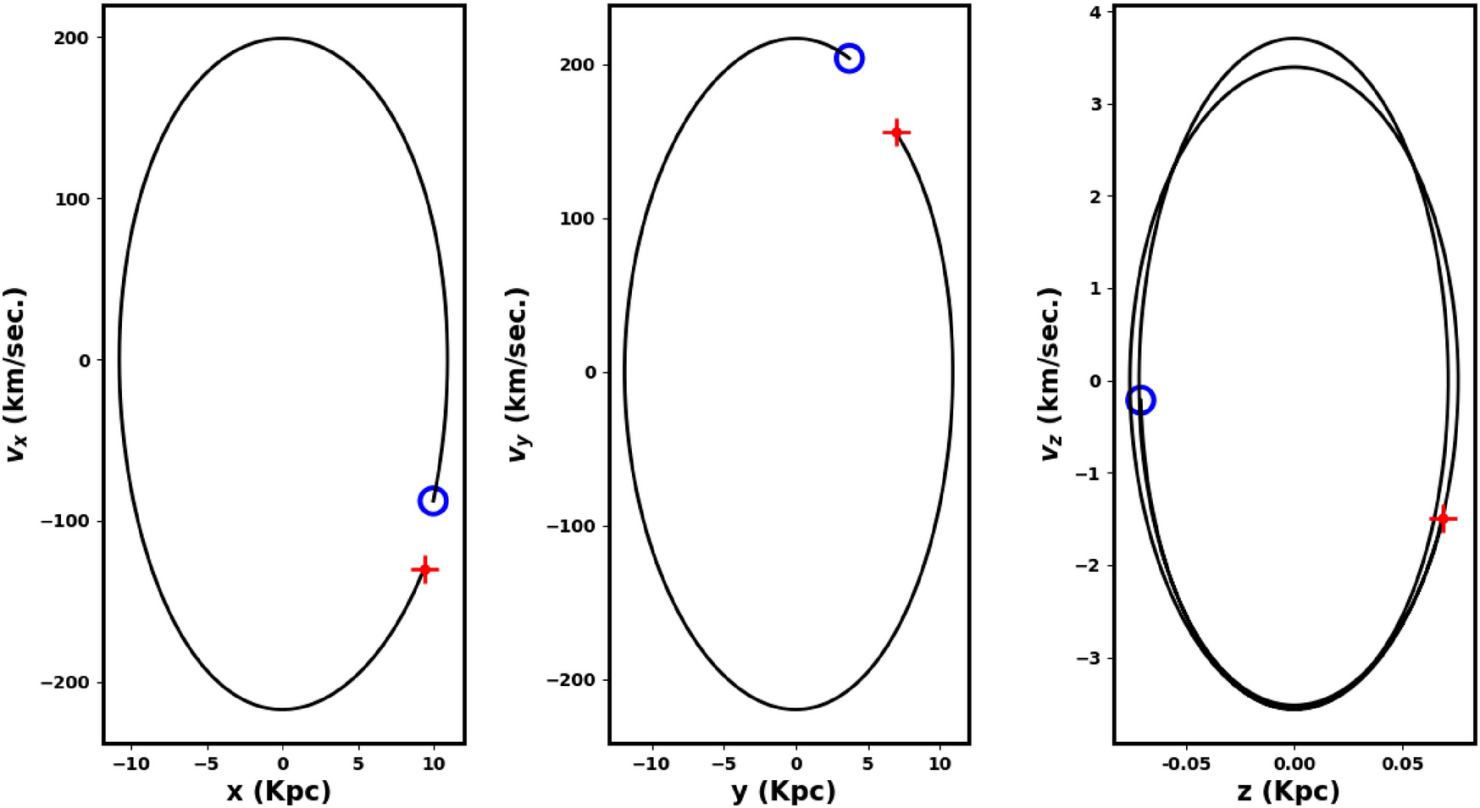
The graph shows the velocities $v_x$, $v_y$, and $v_z$ plotted against the x, y, and z directions, respectively.

The cluster travels parallel to the Galactic plane and in the direction of the Galactic center, see Fig. 15, and the tidal tail is in front of it. In other words, the tidal tail is pointed toward the galactic center. Therefore, the tidal tail could be attributed to the Galactic tidal effects. To better understand this motion, we displayed the velocity of the cluster in different directions with respect to the Galactic center. Fig. 17 shows that the velocity of the cluster is predominantly in the X and Y directions, while in the Z direction (which is perpendicular to the Galactic plane) it is almost zero. These results indicate that the cluster and its tidal tail are nearly moving parallel to the Galactic plane. This result is in consistent with^84^, where they analyze the morphologies of 265 open clusters in Gaia DR2 archive. They concluded that their sample clusters are more elongated in direction parallel to the galactic plane than perpendicular to it, which agrees with^85^. The 3D illustration of the cluster’s motion is shown in Fig. 16. In addition, the subsequent section (Cluster’s Mass Distribution and Segregation) will explore the influence of tidal forces on the mass distribution within clusters and their subsequent effects.

### Cluster’s mass distribution and segregation

The identification of the tidal tail of the King 13 cluster, provides an excellent opportunity to investigate the mass distribution within the cluster and the drift of its stars. Understanding the mass distribution is crucial for studying cluster dynamics, as over time, two-body relaxation causes more massive stars to migrate toward the cluster center, while less massive stars occupy larger volumes and gradually evaporate^86^. This is so-called dynamical mass segregation. This study provides a novel perspective on mass distribution with radius, particularly when considering the tidal tail.

**Fig. 18:**
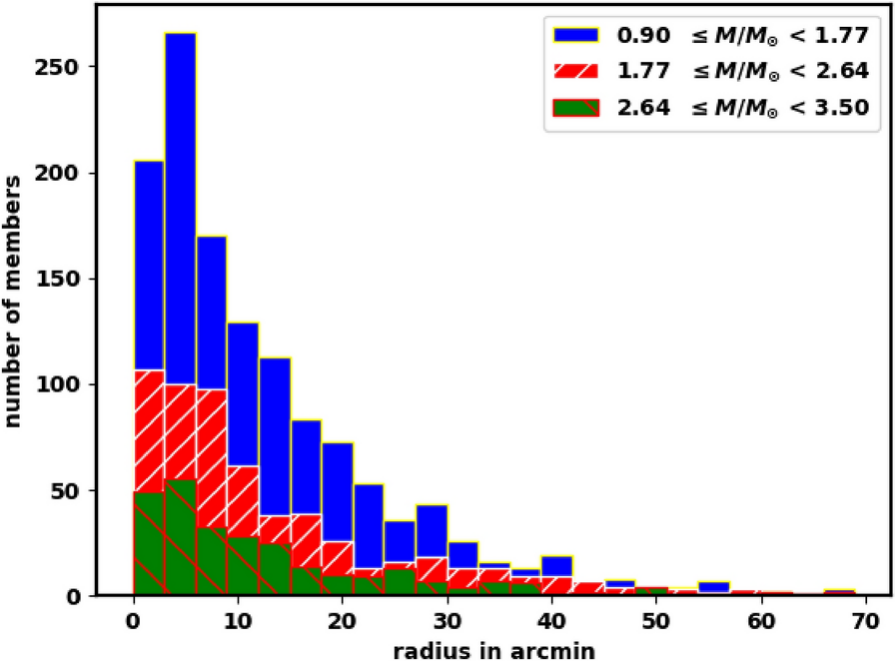
The mass distribution inside King 13 cluster until 70 arcmin about 80.5 pc.

To do so, we divided the member stars into three distinct mass intervals and counted the stars within each radial bin, as illustrated in Fig. 18. The analysis reveals that the mass distribution with respect to radius is nearly identical across all three mass intervals. This finding suggests that the displacement of stars from the cluster is independent of their masses and is instead influenced by their spatial positions within the cluster.

In other words, stars located in the outer regions of the cluster, regardless of their mass, are more likely to drift away until they dissolve into the field, while stars in the core remain tightly bound by the cluster’s gravitational potential and are less affected by Galactic tidal forces. Dynamically, the displacements of member stars are governed primarily by gravitational acceleration rather than their individual masses.

### **Dynamical state**

An essential parameter for understanding the dynamical evolution of a star cluster is the relaxation time. This is the time required for the cluster to lose its initial conditions and for the member stars to approach a Maxwellian velocity distribution. According to^87^, the relaxation time can be described as:

19 $$\begin{aligned} T_R = \dfrac{8.9 \times 10^5 \sqrt{N} \times R_{h}^{1.5}}{\sqrt{m} \times \log (0.4N)} \end{aligned}$$

where *N* is the number of cluster members, $R_h$ is the radius (in pc) containing half of the cluster’s mass, and *m* represents the average stellar mass in solar units.

**Fig. 19:**
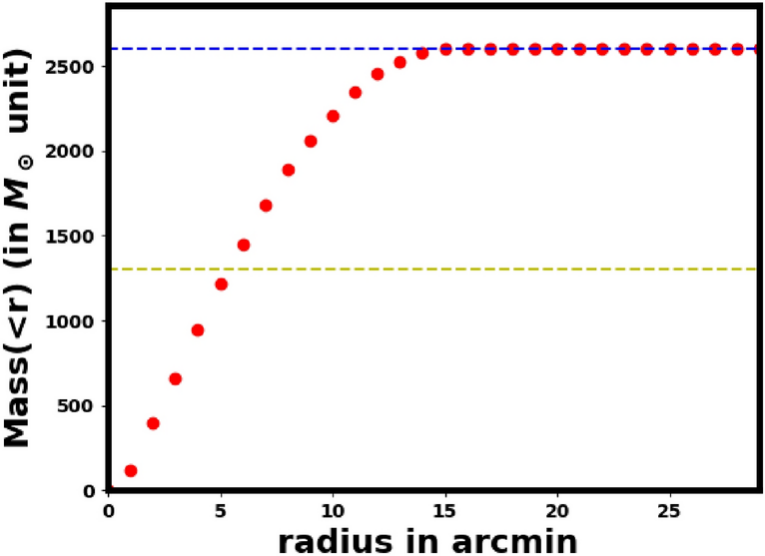
The mass profile $M(<r)$ of King 13. The horizontal blue dashed line indicates the total mass, while the yellow dashed line represents the half-mass radius, $R_h$.

Fig. 19 displays the mass $M(>r)$ as a function of radius *r*. From this, we calculated the half-mass radius, $R_h$, to be 5.4 $\pm$ 0.85 arcmin (about 6.53 pc). Using the above equation, we calculated the relaxation time for King 13 as 134 $\pm$ 13 Myr, which is considerably shorter than the age of the cluster ( 310$\pm$ 28 ). This indicates that King 13 is a dynamically relaxed and stable cluster.

## Summary and conclusions

We performed a detailed study of the young open cluster King 13, using Gaia DR3 photometric and astrometric data, as well as 2MASS data for comparison with Gaia’s color-magnitude diagram (CMD). Our analysis aimed to refine the fundamental parameters of King 13 in the context of the Gaia DR3 era, with a particular focus on its kinematics, dynamics, and structure. The main findings of our study are summarized as follows:

1. To estimate membership, we employed the pyUPMASK Python package along with the HDBSCAN algorithm. *The key focus of this investigation is our new method of evaluating membership probability based on the radius of each shell in the studied cluster, utilizing King model, rather than applying a single probability value to the entire cluster*. Consequently, our analysis yields novel insights into the characteristics of the King 13 cluster. A significant outcome of this methodology is the detection of a dense core accompanied by an elongated halo, aligned with the tangent velocity. Our analysis of the cluster orbit suggested that the cluster moves in the Galactic plane toward the Galactic center direction, and the tidal tail is in front of it; i.e. its tidal tail is directed along the orbital motion. Clearly, the Galactic tidal effects could be responsible for the tidal tail.
2. In King 13, we identified 1571 $\pm$ 41 member stars with a total mass of 2658.4$\pm$ 61.5 $M_{\odot }$. The mass function (MF) for the cluster has been constructed using a step function with two power lows, $\alpha _1$ and $\alpha _2$ , rather than the single power low as suggested by Salpeter. In this cluster, the $\alpha _1$, $\alpha _2$ and $M_{cr}$ are found to be -3.7$\pm$0.4 , 2.3 $\pm$0.15 and 1.25 $\pm$ 0.2 respectively. The high mass slope $\alpha _2$ is near to Salpeter value (2.35). Based on Gaia DR3 data, we estimate the cluster’s age to be 310$\pm$ 28 Myr, and the relaxation time to be 134 $\pm$ 13 Myr, indicating that King 13 is a dynamically relaxed and stable cluster.
3. Our mass segregation analysis of King 13 demonstrates that the radial displacement of stars in the cluster is independent of their individual masses. Instead, the spatial position within the cluster plays a pivotal role: stars in the outer regions are more likely to drift away and eventually dissolve into the field, while those in the core remain gravitationally bound. This indicates that gravitational acceleration, rather than stellar mass, is the primary factor governing the dynamical evolution of member star displacements.
4. The distance modulus of the cluster was determined to be 13.11$\pm$ 1.03 mag, corresponding to a distance of 4187$\pm$ 262 pc. Furthermore, we find that the color excess $E(G_{\text {BP}} - G_{\text {RP}})$ to be 1.17 $\pm$ 0.07 mag. To validate our results, we compared the Gaia CMD with 2MASS data, providing a complementary perspective on the cluster’s photometric properties. The color excess $E(J-K_s)$ and distance modulus are found as 0.44 $\pm$ 0.03 mag and 13.08$\pm$ 0.12 mag, respectively, which correspond to distance 4140 $\pm$ 236 pc.
5. The proper motion components ($\mu _{\alpha } \cos \delta$, $\mu _{\delta }$) and the parallax ($\varpi$) were measured as -2.64 $\pm$ 0.36 mas yr$^{-1}$, -0.89 $\pm$ 0.25 mas yr$^{-1}$, and 0.245 $\pm$ 0.05 mas, respectively. The corresponding cluster distance, derived from the parallax, is 4082 $\pm$ 231 pc, which is in excellent agreement with the photometric data from Gaia and 2MASS, within the associated uncertainties.
6. We also identified 46 member stars with radial velocity data, allowing us to compute the orbital parameters of King 13 using the galpy Python package.

   

In conclusion, in addition to the photometric study, we provide valuable insight into the structure, kinematics, and dynamics of King 13, demonstrating the potential of Gaia DR3 data for open cluster research. The position and mass of open clusters are also important factors in shaping of open clusters. The morphology of open star clusters is clearly influenced by tidal forces and the galactic differential rotation in the galactic plane. These results support the idea that galactic differential rotation and tidal tail play an important role in the evolution of open clusters by stretching the spherical cluster into an ellipsoid and ultimately into a stream in direction of motion or towards to the Galactic center.

## Data Availability

Gaia and 2Mass data : are available for free in webpage https://vizier.cds.unistra.fr/.
